# Single-Walled Carbon Nanotubes (SWCNTs), as a Novel Sorbent for Determination of Mercury in Air

**DOI:** 10.5539/gjhs.v8n7p273

**Published:** 2015-12-16

**Authors:** Farideh Golbabaei, Ali Ebrahimi, Hamid Shirkhanloo, Alireza Koohpaei, Ali Faghihi-Zarandi

**Affiliations:** 1 Occupational Health Engineering Department, School of Public Health, Tehran University of Medical Sciences, Tehran, Iran; 2 Occupational Health Engineering Department, School of Public Health, Qom University of Medical Sciences, Qom, Iran; 3 Iranian Petroleum Industry Health Research Institute (IPIHRI), Occupational and Environmental Health Research Center (OEHRC), Tehran, Iran; 4 Occupational Health Engineering Department, School of Public Health, Kerman University of Medical Sciences, Kerman, Iran

**Keywords:** single-walled carbon nanotubes, adsorption, mercury, air sampling

## Abstract

**Background:**

Based on the noticeable toxicity and numerous application of mercury in industries, removal of mercury vapor through sorbent is an important environmental challenge.

**Purpose of the Study:**

Due to their highly porous and hollow structure, large specific surface area, light mass density and strong interaction, Single-Walled Carbon Nanotubes (SWCNTs) sorbent were selected for this investigation.

**Methods:**

In this study, instrumental conditions, method procedure and different effective parameters such as adsorption efficiency, desorption capacity, time, temperature and repeatability as well as retention time of adsorbed mercury were studied and optimized. Also, mercury vapor was determined by cold vapor atomic absorption spectrometry (CV-AAS). Obtained data were analyzed by Independent T- test, Multivariate linear regression and one way–ANOVA finally.

**Results:**

For 80 mg nanotubes, working range of SWCNT were achieved 0.02-0.7 μg with linear range (R^2^=0.994). Our data revealed that maximum absorption capacity was 0.5 μg g^-1^ as well as limit of detection (LOD) for studied sorbent was 0.006 μg. Also, optimum time and temperature were reported, 10 min and 250 °C respectively. Retention time of mercury on CNTs for three weeks was over 90%. Results of repeated trials indicated that the CNTs had long life, so that after 30 cycles of experiments, efficiency was determined without performance loss.

**Conclusion:**

Results showed that carbon nanotubes have high potential for efficient extraction of mercury from air and can be used for occupational and environmental purposes. The study of adsorption properties of CNTs is recommended.

## 1. Introduction

Environmental and occupational pollution has created many problems in the recent years. Heavy metals are the major pollutants being released from various industrial processes (Zamir, Halladj, & Nasernejad, 2011). Mercury as a toxic heavy metal is created naturally or man-made in the processes such as primary production of metals, gold and cement production, coal burning, oil refining, and chemical synthesis (Brooks, 2009). These situations may leads to substantial contamination of the environment (Tchounwou, 2003; Ligy & Marc 2008). Atmospheric emissions of mercury from human activities are the major known pathway for mercury to enter the air (Selin, 2014). Numerous policy actions have been taken at national and international level over the past several decades to reduce mercury contamination in the environment (N. E. Selin & H. Selin, 2006). This now includes a global treaty to reduce mercury pollution, the 2013 Mina Mata Convention (Kohler, Morgera, Ripley, Schabus, & Tsioumani, 2013). It is global estimated that between 2700 to 6000 metric tons mercury is released to atmosphere early (Streets, Zhang, & Wu, 2009). From toxicological viewpoints, the most important form of mercury is the elemental (Hg^0^) (Luo et al., 2009). Elemental mercury with a high vapor pressure is responsible for frequent intoxication via inhalation (Selin, 2011). The main toxic effects of mercury intoxication are monitored in the kidney and gastrointestinal system (Yard et al., 2012) central nervous system (Carocci, Rovito, Sinicropi, & Genchi, 2014), reproductive system (Martinez et al., 2014) and respiratory system (Luo et al., 2009). Reliable methods for sampling and analysis of elemental mercury in the air were provided by certified resources (NIOSH 6009 and ID-140 OSHA respectively). However the main limitation and disadvantages of these methods are: interference by other compounds, impurities and contamination of reagents in the sample preparation procedures, using acids, time consuming, and possibility of sample loss during preparation. Carbon nanotubes (CNTs) as a new member of the carbon family are cylinder-shaped macromolecules made up of one or more layers of carbon atoms (Ong, Ahmad, Zein, & Tan, 2010). Based on the number of carbon atom layers they may be categorized as single-walled carbon nanotubes (SWCNTs) or multi-walled carbon nanotubes (MWCNTs). Single-Walled Carbon Nanotubes (SWCNTs) were introduced in 1993 by Iijima and Ichihashi (Iijima & Ichihashi, 1993) and Bethune et al. (1993). Carbon nanotubes can be visualized as a sheet of graphite that has been rolled into a tube. In general, the length of SWCNTs may vary from 1 μm to more than 1 mm; whereas, by diameter they may be 0.7 to 1.5 nm (Ong, Ahmad, Zein, & Tan, 2010; Li, Irin, Atore, Green, & Cañas-Carrell, 2013). They have a very broad range of electronic, thermal, and structural properties (Hirlekar, 2009). The adsorption-related applications of CNTs to solve environmental pollution problems have received considerable attention in recent years (Agnihotri, Rood, & Rostam-Abadi, 2005). In some studies, this sorbents have been used successfully for metalcations such as cadmium and copper (Vellaichamy & Palanivelu, 2011; Moradi, Zare, & Yari, 2011) lead (Moradi et al., 2011), chromium (Jung et al., 2013) mercury (Pitoniak, 2003), nickel (Adolph, Xavier, Kriveshini, & Rui 2012), zinc (Vellaichamy et al., 2011; Tajik & Mohammadi, 2011), drugs (Fazelirad, Ranjbar, Taher, & Sargazi, 2015; Wang Sun, Pan, & Xu, 2015) endocrine-disrupting compounds (Jung et al., 2013) and organic contaminants (Yu et al., 2014) in the aquatic environment as well as benzene (Saeedi, Javid, Ghorannevis, Moattar, & Mashinchian, 2014) toluene, dichloromethane and ethanol (Ragunath & Mitra, 2015) in gaseous ambient.

In the present study, a procedure of adsorption of elemental mercury vapor by SWCNTs was provided and the adsorption mode and amount was evaluated and optimized in a gaseous ambient.

## 2. Materials and Methods

In this experimental research, instrumental conditions, method procedure and different effective parameters such as adsorption efficiency, desorption capacity, time, temperature and repeatability as well as retention time of adsorbed mercury were studied and optimized. The atomic absorption spectrometer (GBC) Model (AAS–932 Puls) withhold vapor technique (HG3000) has been used for analysis of samples. Instrumental parameters for cold vapor atomic absorptionspectrometryinall phase’s of the experiments were set as follow: Lamp Current: 4 mA, Wavelength: 253.7 nm, Band pass: 0.5 nm, Argon gas pressure: 400 kPa, Gas flow to the mixer block: 120 mL min^-1^ and Gas flow to the separator: 30 mL min^-1^.

In the first step (according to NIOSH 6009), solutions were prepared at concentrations 1.5, 3 and 6 μgL^-1^. The stationary phase was created containing 40 or 80 mg SWCNTs and was placed in a glass tube with length of 10 cm and the internal and external diameters 4 and 6 mm respectively. Then end-capping with some cotton was performed to prevention of movement the tube contents. Carbon nanotubes were provided by Nano department of oil industry research center, Tehran. Also, all solutions and materials were purchased from Merck Co. Standard solutions with concentrations equal to 1.5, 3 and 6 μg L^-1^ plus argon at flow rate 120 mlmin^-1^ were used to produce various amounts (from 0.006 to 3 μg) of mercury vapor in concentrations 0.1, 0.2 and 3 μgL^-1^ respectively. The amounts of mercury were prepared by continuous flow hydride generator system. The system was designed by peristaltic pump, tin chloride (as reduction agent), ionized water and a mixer. Peristaltic pump rotation rate was set at 90 rpm. Then mercury vapor were pushed across through the SWCNTs by argon and outlet gas were entered in to a quartz adsorption cell. Mercury amount were determined using atomic absorption spectrometry system finally.

## 3. Results

**Figure 1:**
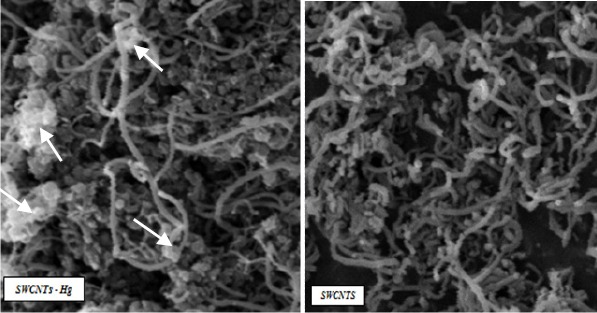
Scanning electron microscopy images of SWCNTs before (right) and after (left) the adsorption of mercury vapors

Figure 1 shows the scanning electron microscopy images obtained from SWCNTs before and after the adsorption of mercury vapors in this research. Adsorption efficiency by 40 and 80 mg of SWCNTs are shown in Table 1.

**Table 1:** Adsorption efficiency for different amounts of sorbent

**40 mg of SWCNTs**				

Mass of mercury (μg)	P-value	Std Deviation	(Mean of adsorption %)	N
0.006	0.884	2.59	96	12
0.012	0.534	4.69	95	12
0.025	0.054	5.87	86.16	12
0.05	0.259	4.93	65.75	12
0.1	0.838	5.01	58.08	12

**80 mg of SWCNTs**				

0.006	-	0	100	12
0.012	-	0	100	12
0.025	-	0	100	12
0.05	-	0	100	12
0.1	0.532	0.9	99.08	12
0.2	0.892	0.77	99.33	12

**Figure 2:**
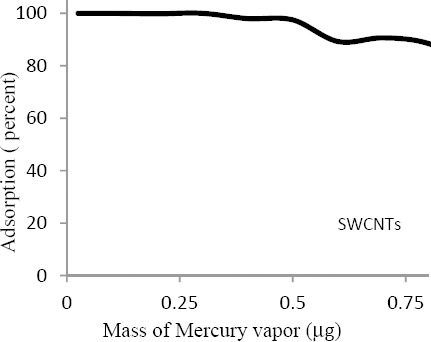
The maximum mass of adsorbed mercury by SWCNTs (n=12)

According to the obtained results, sorbent tube contained 80 mg carbon nanotubes was determined as optimum amount. Furthermore, the results regarding the effects of variables such as the amount of adsorbent, mass of mercury, and various concentrations of mercury on the adsorption rate are summarized in Table 2. Also, results for determination of maximum adsorption amounts of mercury by 80 mg of SWCNTs are shown in Figure 2.

**Table 2:** Effects of studied method variables on adsorption efficiency

Variables	(Φ)	P-value	R^2^
(α): 87.52			
Mass of mercury (μg)			
0.006	-	Reference (0)	
0.012	0.042	0.472	
0.025	-0.074	0.211	
0.05	-0.344	0	
0.1	-0.457	0	0.67
Mass of sorbents (mg)			
80	-	Reference (0)	
40	0.627	0.627	
Mercury concentration(μg/l)			
0.1	-	Reference(0)	
0.2	-0.04	0.496	
0.3	-0.04	0.491	

**Figure 3:**
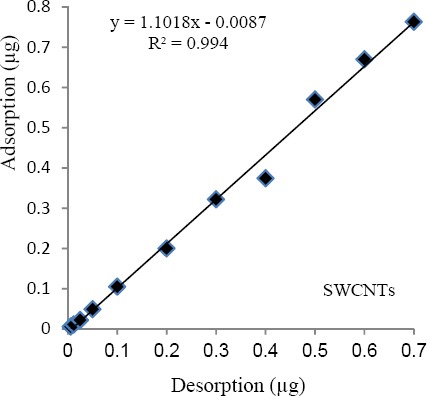
The linear regression between the mean values of the mass adsorption and desorption by SWCNTs (n=6)

For optimization of temperature, the rate of desorption at different temperatures was studied (other parameters were fixed). The maximum desorption rates on the SWCNTs was achieved at the temperature equal to 250 °C. Thus, this temperature was selected as an optimum desorption point. Based on the optimum temperature and doing several experiments, the results showed that the best time for desorption efficiency upper than 90% was about 600-800 seconds with average desorption efficiency equal to 96.31-97.67% (±4.14). Linear regression between the mean values of the mass adsorption and desorption by carbon nanotubes have been shown in Figure 3.

Furtheremore, results indicated that the SWCNT have long life, so that after 30 cycles of adsorption and desorption with 0.1 μg of mercury, efficiency was determined without performance loss as 100%. After adsorption of 0.2 μg mercury on the carbon nanotubes, in order to determination of retention time, the two ends of tubes capped and were kept at zero 0 °C. After various times, carbon nanotubes were desorbed in optimum conditions (250 °C and 10 min) and the results were recorded. Average mercury desorption efficiency after three weeks were determined (105% ± 4.31) finally.

## 4. Discussion

Heavy metals are widely found in the industrial gaseous and aqueous streams. Because of their non-biodegradable, highly toxic nature, they may cause serious problems to human health. Exposure to mercury – even small amounts – may cause serious health problems. The most important problems may be observed after inhalation, ingestion or dermal exposure of different mercury compounds, are neurological and behavioral disorders. Symptoms include tremors, insomnia, memory loss, neuromuscular effects, headaches and cognitive and motor dysfunction. Mild, subclinical signs of central nervous system toxicity can be seen in workers exposed to an elemental mercury level in the air containing 20 μg/m^3^ or more for several years. Kidney effects have been reported, ranging from increased protein in the urine to kidney failure (WHO Media Centre, Mercury and Health, Fact sheet No.361, Updated September 2013).

Many technologies have focused on the efficient elimination of the heavy metals pollutants, such as adsorption, electro dialysis, ultrafiltration, and ion exchange (Pérez-López & Merkoçi, 2012). Among these technologies, adsorption is widely applied, because this method is easy to handle, relatively low cost, and simply equipment. The efficiency of removal adsorption is determined mainly by the adsorption capacity, selectivity for specific compounds, and durability and regenerability of the adsorbents. In recently, the developments of nanoparticles such as CNTs for heavy metal removal have received remarkable attention due to their special features.

In last ten years, a large number (80+) of articles regarding aqueous phase adsorption of a variety of synthetic organic compound (SOC) by CNTs were published in peer-reviewed journals (Apul & Karanfil, 2015) but articles about extraction and separation of occupational pollutant from gaseous ambient aided by CNTs is rare but attractive issue (Saeedi et al., 2014; Ragunath, & Mitra, 2015).

The obtained results showed that the SWCNTs can adsorb mercury vapor in various concentrations successfully. In other studies, the ability of carbon nanotubes in the separathion and removal of metals from aqueous and gaseous environments has been shown (Vellaichamy et al., 2011; Moradi et al., 2011; Adolph Xavier, Kriveshini, & Rui, 2012). Based on the results, cartridge contained 40 mg from SWCNTs cannot absorb the mercury with appropriate level (Table 1). It is revealed that with increasing the amount up to 80 mg, capacity of the sorbent was increased up to 0.5 μg. This phenomenon can be interpreted due to more compact and change in the alignments of the carbon nanotubes, increasing the level and duration of exposure to mercury and differences in physical characteristics such as nanotube diameter (Luo et al., 2009; Vellaichamy & Palanivelu, 2011; Pitoniak, 2003). Therefore, tube contained 80 mg of SWCNTs was selected as optimum point. According to our results, it revealed that between adsorption values by SWCNTs in the various mercury concentrations, there was no significant difference (P_Value_>0.001) (Table 2). It can be concluded that complete adsorption of the various amounts of mercury at different concentrations by SWCNTs, was limited to capacity of carbon nanotubes merely same other researches (Yu et al., 2014; Saeedi et al., 2014).

In general, according to the R^2^ coefficient (R^2^=0.67) and under conditions of the study, it revealed that factors such as the concentration and mass of mercury, the mass and type of the adsorbent have been effectively on the adsorption efficiency by SWCNTs (67%). It seems that other potential factors such as sampling flow, layout of the sorbent tube, length, diameter, surface area, and other features of SWCNTs can influence on the remained adsorption rates (33%) (Saeedi et al., 2014). In the most studies, thermal desorption procedure was applied more than chemical methods (Hirlekar, 2009; Apul & Karanfil, 2015), so in this study, thermal desorption method was selected for desorption and regeneration of sorbent tube.

The optimum point for time and temperature desorption was determined 600–800 seconds and 250 °C respectively. In other studies that designed on the activated carbon and carbon nanotubes, similar to our results, temperature around 100–250 °C and duration of 15–20 minutes for desorption and regeneration process have been reported (Hirlekar, 2009; Vellaichamy & Palanivelu, 2011). Retention time of mercury on SWCNTs, after 21 days at temperature of 0 °C, 105%±4.31 was obtained. This finding is acceptable compared with NIOSH and OSHA standard methods and guidelines (Ligy, Marc, & Deshusses, 2008; NIOSH, 1994).

The detection limit with the mean recoveries (98%±11.80) was equal to 0.006 μg. The quantitative detection limits provided by NIOSH and OSHA for the hapcalite sorbent tubes have been reported 0.03 and 0.02 μg respectively (OSHA, 1998; NIOSH, 1994). Also, according to the experimental conditions of this research, adsorption capacity of SWCNTs was determined as 6 μg per gram of adsorbent. This is in agreement with some other studies showing that, CNTs especially SWCNTs have the higher adsorption capacity (Jahangiri et al., 2011).

In spite of having a lower surface area, they are more efficient adsorbents than activated carbon (Tajik & Mohammadi, 2011). Meanwhile, if in some studies, there are some opposite findings in which activated carbon has higher adsorption capacity than CNFs or CNTs; it is probably due to the mesoporous structure of that specific type of activated carbon (Jahangiri et al., 2011). Under optimal conditions, the use of single-walled carbon nanotube showed good performance, high sensitive and fast sampling of mercury vapor from air.

## 5. Conclusions

Based on our results, Single-walled carbon nanotubes have suitable absorption and desorption capabilities, therefore would be attractive for mercury extraction and separation from the gaseous ambient. Also, this sorbents as ‘materials of the 21st century’ are repeatable and from the viewpoints of economic aspect are affordable. The further study about adsorption properties of CNTs is recommended, because it could shed new light on the mechanism of adsorption in complex systems such as environmental and occupational matrices.
